# Subjective and qualitative assessment of neural disturbance after inferior alveolar nerve transposition for dental implant placement

**DOI:** 10.1186/s40729-016-0047-1

**Published:** 2016-05-14

**Authors:** Fumihiro Nishimaki, Hiroshi Kurita, Shinya Tozawa, Yuji Teramoto, Rishiho Nishizawa, Shin-ichi Yamada

**Affiliations:** Department of Dentistry and Oral Surgery, Shinshu University School of Medicine, 3-3-1, Asahi, Matsumoto-shi, Nagano 390-0804 Japan

**Keywords:** Dental implants, Inferior alveolar nerve, Nerve transposition, Neurosensory disturbance

## Abstract

**Background:**

The purpose of this retrospective study was to accumulate data regarding the quality of postoperative neurosensory function after inferior alveolar nerve (IAN) transposition for dental implant placement.

**Methods:**

The study included seven consecutive patients who underwent IAN transposition surgery for the insertion of a dental implant into the atrophic posterior mandible. Of these, six patients (seven sides) were available for long-term assessment of postoperative IAN function. Neurosensory disturbance of the IAN was assessed objectively using the modified SW perception test reported by Semmes and Weinstein. In addition, the quality of nerve paralysis was assessed according to the criteria reported by Highet.

**Results:**

The median follow-up time was 49 months (range 12–105 months). No implant loss was observed during the follow-up. All patients experienced numbness immediately and the days after surgery. Complete recovery of neural function was observed on two sides; weak hypoesthesia was observed on two sides, moderate hypoesthesia on two sides, and severe hypoesthesia on one side. However, only one patient expressed concern about IAN function.

**Conclusions:**

IAN transposition is a useful method for placing implants in the atrophic posterior mandible. However, the procedure is complicated, with the possibility of some degree of neurosensory disturbance, although in most of our cases, it resolved within a clinically acceptable period.

## Background

Tooth loss is one of the common causes of reduced quality of life in adults. Dental implants have become a widely accepted treatment option for both partially and completely edentulous patients [1–3]. However, in cases of posterior mandibular atrophy, suitably sized implants cannot be placed without encroaching on the inferior alveolar nerve (IAN). In such cases, restorative options include the use of short fixtures, onlay bone grafting to increase the ridge height, and more complicated and detailed imaging studies to allow positioning of implants alongside and not in the nerve canal during the procedure [4]. Another option is to displace the IAN laterally from its canal during implant insertion (nerve lateralization, transposition, or nerve reposition) [5, 6]. The advantages of IAN lateralization include the ability to place longer fixtures and to engage two cortices for initial implant stability [3]. However, as a possible complication of the procedure, temporary or permanent disturbance of the neurosensory function of the IAN is common. The risk of IAN morbidity sometimes results in the limited use of this procedure. However, only few studies have evaluated the long-term results of neurosensory disturbance (ND) following IAN lateralization for dental implant placement in the atrophic edentulous mandible, and the range and quality of the neurosensory function of the IAN have not been fully analyzed [7–10].

The purpose of this retrospective study was to investigate the quality of postoperative neurosensory function after IAN transposition for dental implant placement.

## Methods

**Fig. 1:**
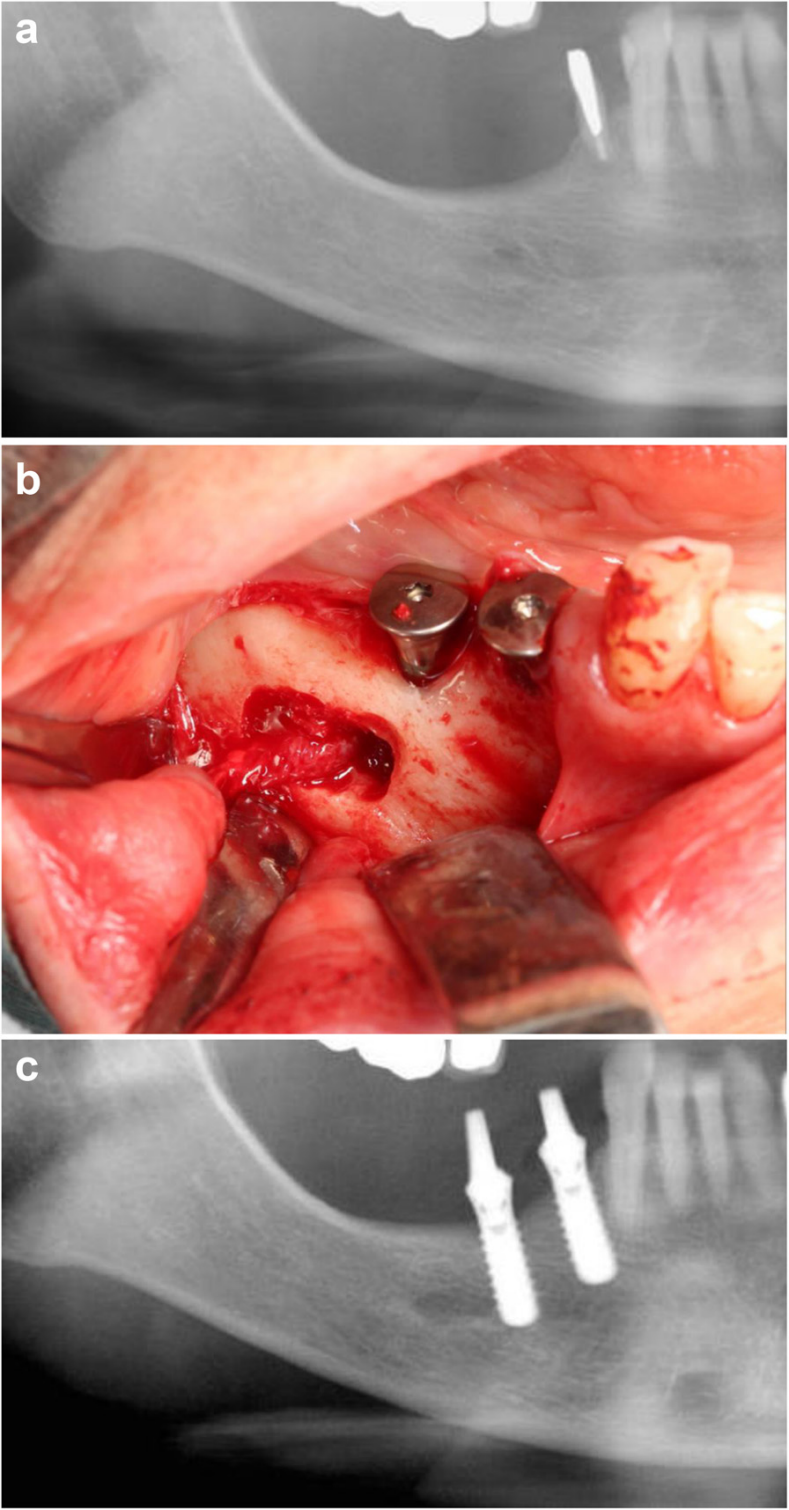
Alveolar nerve repositioning in a partially edentulous mandible. **a** Preoperative radiograph. **b** The inferior alveolar nerve was transposed from the mental foramen. **c** Postoperative radiograph after implant insertion

This study was conducted in compliance with the principles of the Declaration of Helsinki, and was approved by the Committee for Ethics at Shinshu University School of Medicine. Patients who underwent dental rehabilitation by insertion of dental implants between 2000 and 2012 in our hospital were reviewed. Of these, seven patients underwent transposition of the IAN for dental implant placement and thus included in this retrospective assessment. These included six women and one man, with a median age of 64 years old (range 38–75 years old). The same surgical procedure, IAN transposition, was performed in each patient. All operations were done by same operator (H.K.). A crestal and anterior releasing incision was performed for visualization of the entire mental foramen and the lateral aspect of the mandible. The IAN was then extracted by gentle carving of the cortex and cancellous bone around the mental foramen and lateral wall of the inferior alveolar canal using a bone-cutting burr. The IAN was exposed and gently and minimally deflected laterally from the mental foramen to a distance 3–5 mm posterior to the most distal implant using a blunt curette. The incisor branch was cut in all cases. The implants could then be placed under direct visualization. After implant placement, the IAN was repositioned in the osseous canal against the implants. The mucoperiosteal flap was repositioned, and complete closure was achieved (Fig. 1). Implant exposure was performed 3 months after the surgical procedure, and prosthetic rehabilitation began thereafter.

**Fig. 2:**
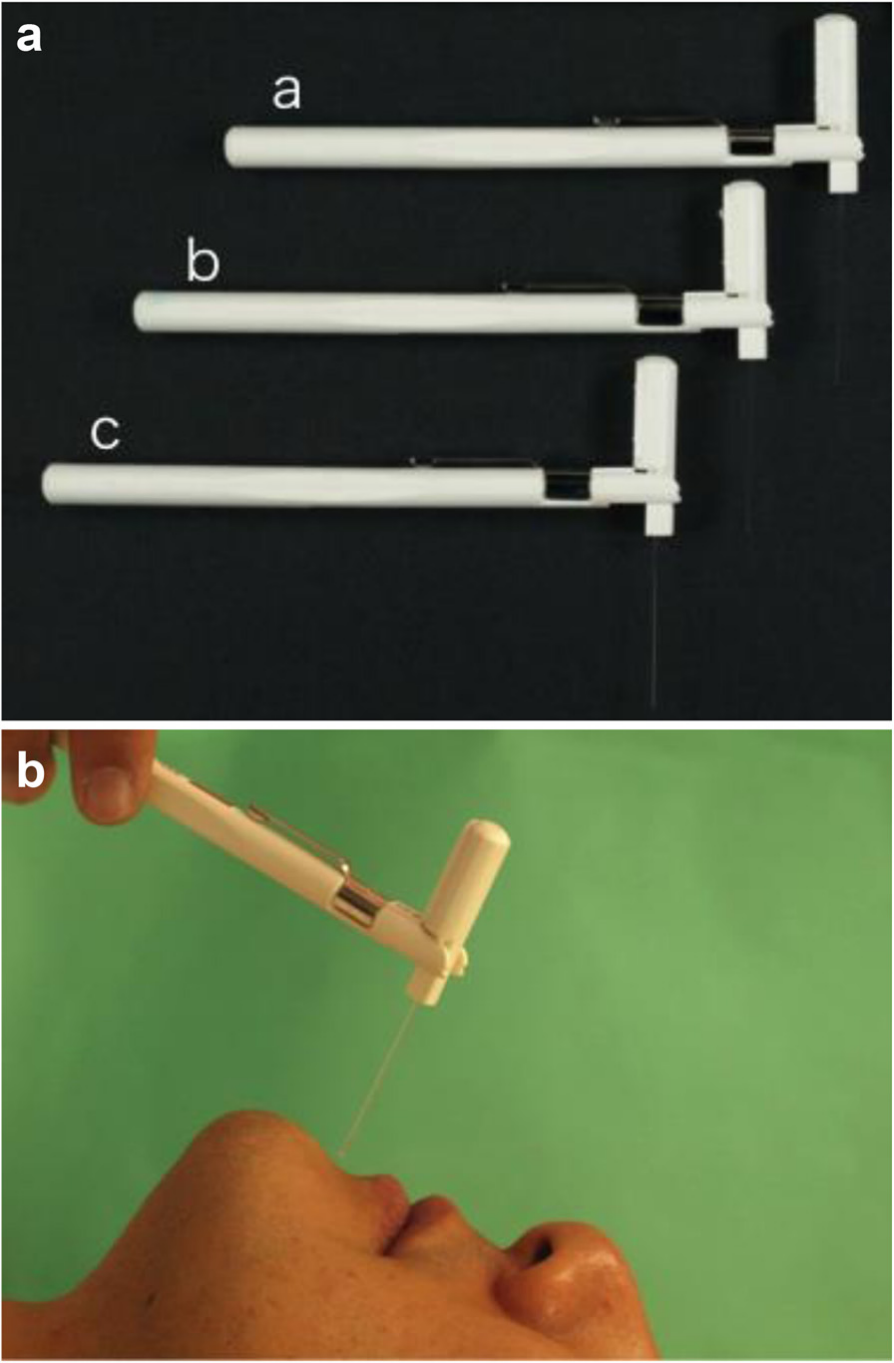
**a** SW perception tester is composed of different diameters (*a*: 0.165 mm, *b*: 0.215 mm, *c*: 0.315 mm). **b** The use of SW perception tester started from a filament of 0.165 mm in diameter of the most weak force, and performed three times at one site

**Fig. 3:**
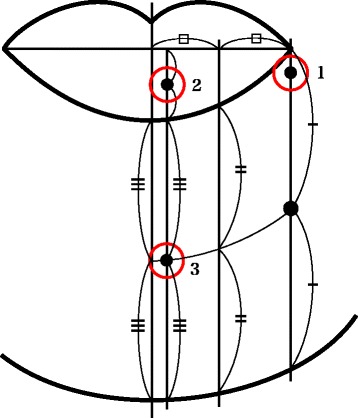
Site of evaluation. *(1)* Corner of the mouth: 5 mm below the corner of the mouth. *(2)* Lower lip: 5 mm laterally from the midline. *(3)* Mental region: at the midpoint of the perpendicular from the lower edge to the lower lip to the chin and 5 mm laterally from the midline

The patients were recalled for evaluation of the function of the IAN in the range between 12 and 105 months (median follow-up time 49 months). We performed a relatively objective assessment of mental nerve paralysis by applying the modified SW perception test reported by Semmes and Weinstein [11, 12]. The presence or absence of sensation was tested with three nylon monofilaments of the same length but different diameters (0.165, 0.215, and 0.315 mm) (Fig. 2). Three representative points (labial commissure, lower lip, and mental region) were assessed (Fig. 3). According to Wernor et al. [13], whether the patients, with closed-eyes and in a horizontal position, could be aware of the stimulation was assessed in a quiet environment. The filament was pressed to the assessment points, and maintained for a few second. The use of SW perception tester started from a filament of 0.165 mm in diameter of the most weak force, and performed three times at one site. The threshold of the tester raised with filaments of 0.215 and 0.315 mm in diameter sequentially. Each point was tested three times separately by the same examiner and at the same pressure, and the presence of sensation was judged if the patients correctly expressed positive sensation more than twice. The presence of sensation was scored as 1 point, and total function of the IAN was graded by adding the scores from 0 to 9 points (3 points × 3 different diameters of nylon monofilaments). In addition, the quality of nerve paralysis was assessed according to the criteria reported by Highet [14] (Table 1).

**Table 1:** Highet grading

Stage 0	Complete sensory loss
Stage 1	Advent of deep pain
Stage 2	Some degree of tactile recovery and pain on superficial skin
Stage 2+	Emergence of hyperalgesia and complete tactile recovery and pain
Stage 3	Recovery of tactile sensation without pain; hyperalgesia disappearance
Stage 3+	Restoration of position sense, to some extent, with 2-point discrimination (2PD: 6–15 mm)
Stage 4	Full sensory recovery (2PD: 2–6 mm)

## Results

In total, eight IAN transposition procedures were performed in seven patients. One patient underwent bilateral surgery. Surgery was performed under general anesthesia in four patients and under local anesthesia in three patients. The IAN was lateralized for a four-tooth breadth on one side, three-tooth breadth on three sides, and two-tooth breadth on three sides. In total, 22 dental implants were placed, with an average of 3.1 implants per side (three implants in six sides and two in two sides). The residual bone height above the IAN ranged from 1 to 10 mm, with an average of 7.43 mm (SD 1.50 mm). The mean implant length was 12.77 mm (11 mm for two implants, 12 mm for five, 13 mm for 13, and 15 mm for two); the mean implant diameter was 4.45 mm (4.1 mm for five implants, 4.5 mm for 15, and 5 mm for two). All patients experienced numbness immediately and the day after surgery. All patients received short-term corticosteroid therapy(dexamethasone, Decadron^®^, 6.6 mg/day) within 1 week to reduce the postoperative swelling and compression of IAN, and six patients received oral vitamin B12 (methylcobalamin, Methycobal^®^, 1500 μg/day) for less than 6 months to facilitate the restoration of IAN function.

The results of our assessment of IAN function are summarized in Table 2. One patient did not respond to the recall; thus, only six patients (seven sides) were available for assessment. The median follow-up time was 49 months (range 12–105 months). No implant loss was observed during the follow-up.

**Table 2:** Results of assessment of sensory neural function after inferior alveolar nerve (IAN) transposition

No.	Sex	Age (years)	Range of IAN lateralization (width)	Implant site	Follow-up period (months)	Scores on the SW test	Grade of neurosensory disturbance (Highet grading)
1	Female	64	2 teeth	34, 35, 36	Lost	–	–
2	Female	71	2 teeth	43, 45, 46	105	1	2
3	Female	59	3 teeth	35, 36, 37	53	7	3+
			3 teeth	45, 46, 47		9	4
4	Female	68	3 teeth	45, 47	49	8	3+
5	Female	38	3 teeth	43, 44, 46	13	3	3+
6	Male	64	4 teeth	43, 44, 46	12	9	4
7	Female	75	2 teeth	44, 45	13	8	3+

Complete recovery (9 points in SW score and stage 4 in Highet grading) of neural function was observed on two side; weak hypoesthesia (7–8 points in SW score and stage 3+ in Highet grading) was observed on two sides, moderate hypoesthesia (3–6 points in SW score and stage 3+ in Highet grading) on two sides, and severe hypoesthesia (1 point in SW score and stage 2 in Highet grading) on one side. At the time of recall, three patients (four sides) were not concerned about IAN function, whereas two patients felt a slight disturbance and one patient complained of neurosensory disorder.

## Discussion

IAN reposition may serve as a viable treatment option in the severely resorbed mandibles. Repositioning is performed via one of the two surgical techniques, lateralization, or transposition, with lateralization yielding lower degrees of nerve deficiency. In lateralization, the IAN is exposed and retracted laterally, held in this position during implant placement, then released to rest against the implants [15]. In the transposition technique, the mental foramen is included in the osteotomy, to allow incisive branch excision, so that the IAN can be pulled into a new position, generally more posterior [16]. The advantages of IAN transposition include the ability to place longer fixtures and to engage two cortices for initial stability [3]. Further, implant insertion can occur immediately; there is no need for long waiting periods or other surgical donor sites that is sometimes required in techniques such as bone augmentation and alveolar bone distraction.

Jensen and Nock were the first to report an IAN transposition for the placement of osseointegrated implants in the posterior mandible area [5]. However, this surgical procedure involved the inherent risk of ND of the IAN. Hypoesthesia, paresthesia, and hyperesthesia are the most common postoperative complications after IAN lateralization, as observed with any surgery where a peripheral nerve is moved from its physiological site. In this study, the breadth of IAN lateralization was not associated with the occurrence of ND significantly. Some studies have evaluated the prevalence of ND after IAN lateralization surgery. Ferrigno et al. reported total ND of 21.1 % and normal neurosensory function of 73 % after 6 months of surgery [7]. Rosenquist reported that 77 % patients had no ND after 6 months of surgery and 94 % patients were normofunctional after 18 months [8]. Hashemi prospectively investigated the types and durations of ND relative to IAN lateralization and found that all patients reported ND in the first week, decreasing to 26 % at the end of the first month, and 3 % at the end of the sixth month, with no changes at the end of 1 year [9]. Fernandez Diaz and Naval Gias utilized a piezotome in IAN lateralization surgery and reported good results, with an IAN normofunctional rate of 94.7 % at 8 weeks after surgery [10]. B.M. Vetromilla reported that the patients who underwent transposition, neurosensory alterations were observed in 58.9 % of patients initially, and the condition remained for 22.1 % of those affected at the end of the study [17]. The results of these studies suggest that the risk of ND after IAN transposition or lateralization is low. However, in the present study, complete recovery of neural function at more than 1-year follow-up was observed only on one operative side, and the other patients (six of seven operative sides) reported at least a weak disturbance of IAN sensory function when evaluated by the relatively objective method.

Although the previous studies reported good results concerning ND in IAN transposition surgery, the methods for evaluating ND differed, and most of the studies did not fully describe the evaluation procedure. The evaluation of ND of the IAN can be performed by purely subjective (questionnaire), relatively objective (static light touch, 2-point discrimination, etc.), and purely objective methods (trigeminal somatosensory evoked potential, blink reflex method, etc.). It is well known that there are discrepancies in the assessment results for nerve impairment between the subjective and objective methods. In the present study, only one patient complained of neurosensory disorder, while five out of six patients had ND if assessed by the objective method (SW test and Highet grading).

In cases where ND was judged by clinical assessment, the presence or absence of ND would be influenced by the evaluation criteria. In the studies by Rosenquist, Ferrigno et al., and Fernandez Diaz and Naval Gias, a patient who presented a 2-point discrimination capability below 14 or 15 mm was considered normofunctional [7, 8, 10]. In our study, if the patient was assessed according to the same criteria (Highet grading of ≥3+), six out of seven (85.7 %) operative sides were considered normofunctional. Our results showed ND rates after IAN lateralization similar to those reported by the studies described above. In the present study, the quality of ND was evaluated by Highet grading; according to this grading, complete recovery (grade 4) was obtained only on two operative sides and weak hypoesthesia (grade 3+) was observed on four operative sides. These results suggest that although the neurosensory function of the IAN was restored to almost normal levels over a period of time after IAN lateralization surgery, a weak or negligible degree of ND remained in some patients, which could be identified only by objective evaluation methods.

In this study, although two patients reported slight disturbance and one patient complained of ND, all patients were satisfied with the results of restoration by dental implant insertion. Hashemi reported that in his study, 82 of 87 patients were satisfied with the results of nerve lateralization after 1 year [9]. It was suggested that subjective reports of perceived sensory changes are initially overestimated, but may be underestimated as the postoperative time interval increases. It is possible that patients adapt or become accustomed to what they consider “normal” over time [15]. It is possible that a weak impairment of IAN function remains after IAN transposition in some patients. However, the impairment is negligible, and the patients may become accustomed to it.

Dental restoration by means of dental implants can provide good functional rehabilitation, particularly in patients with atrophic mandibles. IAN lateralization is a useful method for placing implants in the atrophic posterior mandible. However, there is a possibility of the neurosensory function of the IAN being disturbed, although in most cases, it resolves within a clinically acceptable period.

Piezosurgery is a recently developed system for cutting bone with microvibration [18]. The device cuts mineralized tissue exactly and smoothly, while adjacent soft and nerve tissues remain unharmed because of the cessation of surgical action when the scalpel comes into contact with non-mineralized tissues [19]. The technique has shown to be feasible in IAN transposition with the advantages of smaller osteotomies and preservation of the vascular-nervous bundle [20]. The longer time required for the operation has been reported as a disadvantage [21].

As another treatment options against the vertical discrepancy of the alveolar ridge, the placement of short implants has performed with high success rate [22]. Nevertheless, it is pointed out that biomechanics is related to the denture design, while is directly associated with the mean rates of success and failure, and the use of short implants and dentures with excessive lever arms is a factor for failure [6, 7].

## Conclusions

In conclusion, we investigated the quality of postoperative neurosensory function after IAN transposition for dental implant placement. IAN transposition is a useful method for placing implants in the atrophic posterior mandible. However, the procedure is complicated, with the possibility of some degree of neurosensory disturbance, although in most of our cases, it resolved within a clinically acceptable period.
